# Breaking down glioma primary cilia disassembly

**DOI:** 10.1093/noajnl/vdaf217

**Published:** 2025-10-10

**Authors:** Matthew R Sarkisian, Loic P Deleyrolle, Joshua J Breunig

**Affiliations:** Department of Neuroscience, McKnight Brain Institute, University of Florida College of Medicine, Gainesville, Florida (M.R.S.); Preston A. Wells Jr. Center for Brain Tumor Therapy, University of Florida College of Medicine, Gainesville, Florida (M.R.S.); Department of Molecular Medicine, Mayo Clinic, Jacksonville, Florida (L.P.D.); Board of Governors Regenerative Medicine Institute, Cedars-Sinai Medical Center, Los Angeles, California (J.J.B.); Department of Biomedical Sciences, Cedars-Sinai Medical ­Center, Los Angeles, California (J.J.B.); Center for Neural Sciences in Medicine, Cedars-Sinai Medical Center, Los Angeles, California (J.J.B.); Cedars-Sinai Medical Center, Samuel Oschin Comprehensive Cancer Institute, Los Angeles, California (J.J.B.)

**Keywords:** AURKA, cancer stem cells, cilia, glioblastoma, HDAC6

## Abstract

While many postmitotic cells in the body harbor cilia, certain aggressive cancers such as glioblastoma (GBM) display low frequencies of cells harboring a primary cilium. Ciliated GBM cells that plan to multiply have to disassemble their cilium in order for centrioles to duplicate and re-purpose for mitosis. Little is known about the molecular mechanisms underlying cilia disassembly in GBM, or whether this may represent a driving factor in disease onset, progression, or recurrence. In many cell types, ciliary disassembly is thought to be orchestrated by the aurora kinase A (AURKA) and histone deacetylase 6 (HDAC6) signaling axis. These molecules are often overexpressed in GBM, perhaps owing to the less frequent observation of ciliated GBM cells. Here, we review regulators of the core pathway, and discuss recent studies attempting to inhibit AURKA and HDAC6 in patient and mouse models of GBM and resulting effects on cilia. In the face of potent inhibitors, GBM cells appear to engage pathways independent of the core axis to promote cilia disassembly and/or engage other forms of modified axonemal tubulin to ensure persistence of cilia on GBM cells. GBMs upregulate a host of proteins implicated to drive cilia disassembly. Thus, clarifying these alternate mechanisms may be important as the roles of cilia in tumor formation and propagation, angiogenesis, and treatment resistance are increasingly reported. A deeper understanding of the role of cilia in these hallmarks of glioma may hold clues to the high recurrence rate of GBM.

Key Points

- The regulation of cilia disassembly in all aspects of GBM is poorly understood.
- Many proteins that drive cilia disassembly are upregulated in GBM.
- New therapeutic inhibitors/targets are needed to lock GBM cells in their ciliated state.

Certain biological structures undergo remarkably complex, rapid, and repeated disassembly and reassembly—for example, the deconstruction of dendritic spines or synaptic densities during neural development and plasticity.^1^^,^^2^ Similarly, most eukaryotic cell types must disassemble their primary cilium during cell proliferation. Primary cilia are hair-like organelles that elongate from the cell’s mother centriole and protrude from the cell body into the cell’s microenvironment. Primary cilia have microtubule-based axonemes that serve as a scaffold for intraflagellar transport (IFT), a process that shuttles ciliary content to and from the ciliary tip. Primary cilia are typical features of most differentiated or quiescent (G0) cells (Figure 1). However, during the cell cycle, primary cilia must begin disassembling during S phase so that centrioles can properly duplicate and re-purpose for mitosis (M-phase), after which the cilium reassembles from the mother centriole in G1, becoming known as the basal body. Generally, cilia disassembly is characterized by resorption back into the cell (probably more common), or thru actin-based contraction that “pinch off” or excise portions or large chunks of cilia (Figure 1). The reader is referred to a number of excellent and highly detailed reviews on molecular mechanisms of cilia assembly and disassembly.^3-9^ Whether disrupted cilia disassembly mechanisms play a key role in the initiation or maintenance of aggressive brain tumors such as glioblastoma (GBM) has not been extensively investigated.

**Figure 1.**
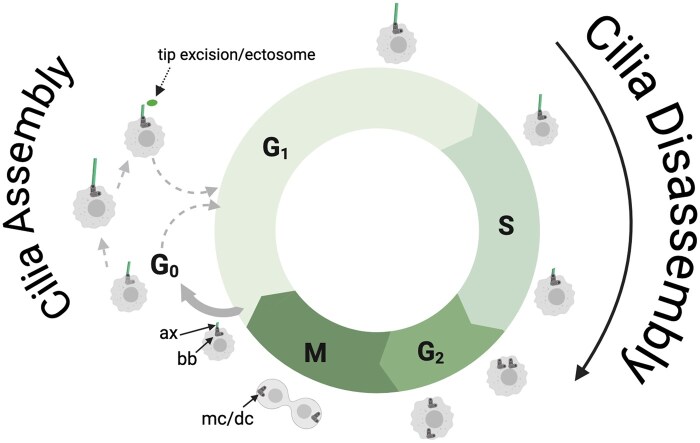
Cilia assembly and disassembly during the cell cycle. After M-phase, primary cilia form in G0 or G1, and begin disassembling during S into G2. During G0, cilia are resorbed back into the cell or undergo actin-based excision promoting cell cycle re-entry. bb= basal body, ax = axoneme, mc/dc = mother centriole/daughter centriole. Image created with BioRender.com.

GBM, a grade IV astrocytoma, is considered amongst the most aggressive and lethal human cancers. GBMs putatively derive from some type of glial progenitor or neural stem cell type.^10-13^ In the developing brain, radial glia produce the majority of neurons, astrocytes and ependymal cells in the postnatal brain which are all ciliated^14^ (Figure 2). Radial glial cells derive from neuroepithelial cells, both of which enrich the regulatory GTPase, ARL13B in their cilium.^15^ Deletion of ARL13B, which causes cilia loss across cell types,^16^ can significantly alter neurogenesis depending on when it is targeted. Deleting ARL13B in neuroepithelial cells which give rise to radial glia causes radial glial scaffolds to collapse and lose polarity to the point cells abnormally form rosettes of prematurely differentiated cells along the ventricular surface.^15^ Astrocytes in the adult brain possess cilia, but little is known about the role of cilia during astrocyte development or in mature brain. In vitro, ablating the cilia can promote astrocyte proliferation.^17^ Recently, using mouse models to target cilia on astrocytes, ablation early in development appears to slow their proliferation, whereas depletion in mature brain appears to expand their morphological structure.^18^ Further deleting ARL13B throughout the brain at P14 and examination at P60 revealed no visible defects in the brain, though it is unclear if aged mice eventually develop tumors. Other studies that induced widespread depletion of cilia, in adult brain, for example, thru conditional loss of *Kif3a* or *Ift88*, have not reported rapid induction of brain tumors.^19^^,^^20^ Mature oligodendrocytes appear to permanently disassemble their cilium, but retain their cilium during premyelinating phases (Figure 2). Ablation of oligodendrocyte progenitor cilia slows proliferation.^21^^,^^22^ Taken together, the loss of cilia does not seem to immediately impart a glioma phenotype, though it is not clear if sustained expression of these mutations in the animal would lead to transformed cell types (Figure 2). It is hypothesized that one source of gliomas could be glial progenitors that reside along the subventricular (SVZ) surface in adult.^23^ There are two types of astroglial progenitors in the SVZ of adult. B1 cells whose cilia extend into the ventricle, and recently characterized B2 cells that do not contact the ventricle and are ciliated.^24^ Whether failure to maintain these cilia could ultimately trigger malignant transformation is unknown as loss of primary cilia in differentiating cells is implicated in various pathological states.^9^ As described below, many GBMs appear to abnormally express or upregulate proteins that drive cilia disassembly, but whether this precedes or follows tumor initiation is unknown.

**Figure 2.**
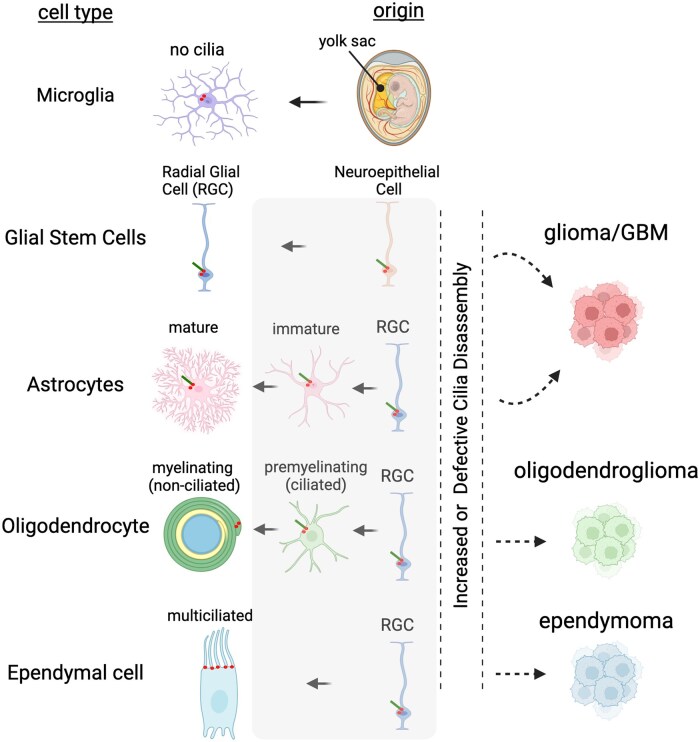
Glial-derived cell types in the developing and mature brain undergo cilia assembly and disassembly. Originating in the yolk sac, microglia in the brain are believed to completely lack primary cilia. Neuroepithelial cells, which give rise to radial glial cells (RGC), are chief neural stem cell types that assemble and disassemble their cilia during embryogenesis. RGCs give rise to astrocytes which grow primary cilia. RGCs give rise to oligodendrocytes which before they become myelinating display cilia but disassemble their cilia during myelinating phases. RGCs also produce multiciliated ependymal cells that line the SVZ surface. The shaded region represents potential vulnerable periods where it is unknown if increased, failed, or aberrant cilia disassembly during maturation of each of these cell types is a contributing factor to various tumor types. Image created with BioRender.com.

Compared to ciliogenesis/cilia assembly, it is generally agreed that the mechanisms controlling ciliary disassembly are less understood.^7^^,^^25^^,^^26^ Notably, many cancers display a signature of primary cilia loss, and defects in ciliary disassembly have been associated with various cancers.^27^^,^^28^ Indeed, tumor cilia frequencies are typically reduced in high grade glioma (HGG) such as GBM.^29-32^ Whether and how the residual cilia/ciliated cells contribute to tumor growth and treatment resistance is not the focus of this review. Rather, the extent of mutations or whether or not cilia disassembly is typically defective in glioma has not received much attention. Similarly, few studies have addressed whether gliomas can be arrested in their ciliated state by interfering with the cilia disassembly process.

Here we discuss briefly some of the core axis molecules thought to be involved in primary cilia disassembly. Due to a paucity of studies aimed at inhibiting cilia disassembly in glioma, this review is somewhat restricted to our group’s recent studies that attempted to target or inhibit some of these players in mouse and human glioma cells and unexpected changes when targeting cilia disassembly. We discuss various directions, unanswered questions, and considerations around the significance of altered cilia disassembly in GBM cells.

## The Core Axis of AURKA and HDAC6 in Primary Cilia Disassembly

**Figure 3.**
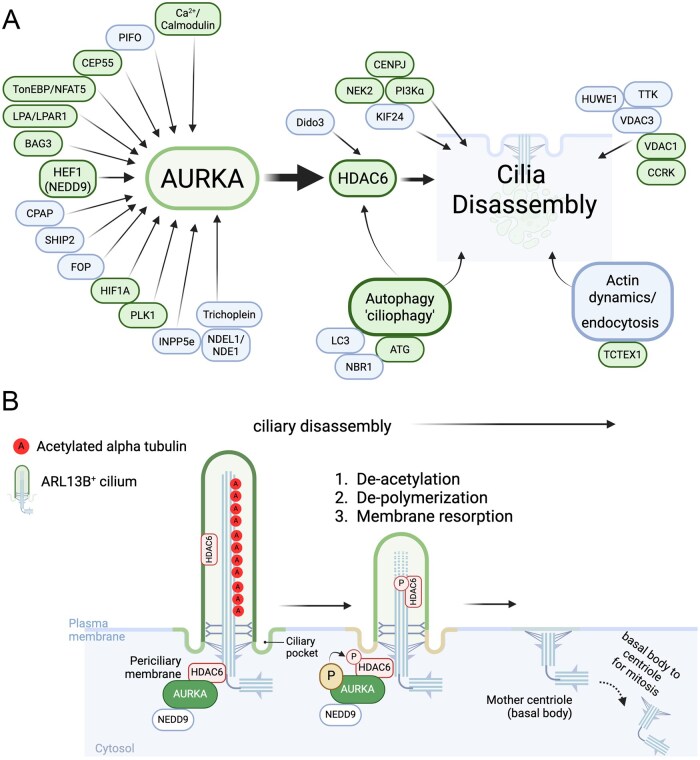
Aurora Kinase A (AURKA) axis of cilia disassembly. (A) Various direct regulators of AURKA that drive ciliary disassembly thru HDAC6. Also indicated are other pathways that appear independent of AURKA or act on HDAC6 to promote cilia loss. Genes surrounding AURKA/HDAC6 that are reportedly upregulated in glioma are shaded green. (B) Many ARL13B^+^ primary cilia possess acetylated microtubules. Upon activation of AURKA by NEDD9/HEF1, HDAC6 becomes phosphorylated and deacetylates the microtubules leading to shortened and ciliary resorption. If dividing, centrioles must detach and migrate to spindle poles for mitosis. Image created with BioRender.com.

Much of our basic understanding of ciliogenesis and cilia function, for example, discovery and initial characterization of IFT, comes from studies of the single cell, green algae *Chlamydomonas*.^33-35^ Indeed, *Chlamydomonas* aurora/Ipl1p-like protein kinase (CALK), was first described to have a role in destabilizing and promoting cilium/flagella resorption.^36^^,^^37^ But how CALK was regulated and its downstream targets that ensure cilia disassembly at the time was unknown. Using hTERT-RPE1 cells, Pugacheva and colleagues (2007) later laid foundation for this in higher eukaryotes. They showed that HEF1(aka NEDD9), a scaffolding protein highly upregulated in various cancers including GBM,^38^ activated Aurora Kinase A (AURKA), which is distantly related to CALK, at the ciliary base.^39^ HEF1 activation of AURKA induced phosphorylation of HDAC6, which then localized to cilia where it de-acetylated microtubules, presumably destabilizing them and promoting collapse or resorption of the ciliary microtubular backbone. Moreover siRNA-mediated knockdown of AURKA or inhibitors of HDAC6 (eg trichostatin-A, tubacin) served to stabilize the cilium and inhibited resorption.^39^ Since these discoveries, a host of pathways impinging on AURKA and HDAC6 have been identified that promote ciliary disassembly and forming a core axis of ciliary disassembly in most cell types ­(Figure 3A).^39-55^

Other pathways that circumvent AURKA during cilia disassembly have been noted.^56-60^ For example, the interaction between Nek2 and KIF24 may promote cilia disassembly independent of AURKA, and function to suppress cilia assembly while cells are proliferating.^61^ It is also known that actin dynamics within the cell body (eg Tctex1)^62^ or are recruited into the cilia axoneme can lead to cilia resorption or excision.^63^^,^^64^ Upon stress conditions, cilia may also be consumed thru autophagic mechanisms, as autophagy proteins have been shown to localize to the cilia base and axoneme.^65-67^

Overall, the triggering of core axis pathways appears to lead to conserved sequence of events that regulate the process of cilia disassembly (Figure 3B). First is general activation of AURKA and deacetylation of microtubules, presumably thru HDAC6. Second is depolymerization of microtubules within the ciliary axoneme. Third the ciliary membrane remodeled and resorbed back into the cell. During this process the cell must also use molecules such as KIF24 to ensure cilia assembly/re-assembly is inhibited.^25^

AURKA and HDAC6 are frequently overexpressed in GBM and implicated in driving tumor growth.^68-73^ Considering their activity appears at the center of ciliary disassembly may explain the lower frequency of cilia observed in GBM.^31^ Thus, below we summarize studies from our group and others that made attempts to see if blocking AURKA and HDAC6 activity in GBM cells could “freeze” tumor cells in their ciliated states while inhibiting GBM cell proliferation.

## Inhibiting AURKA in Glioma Cells

**Figure 4.**
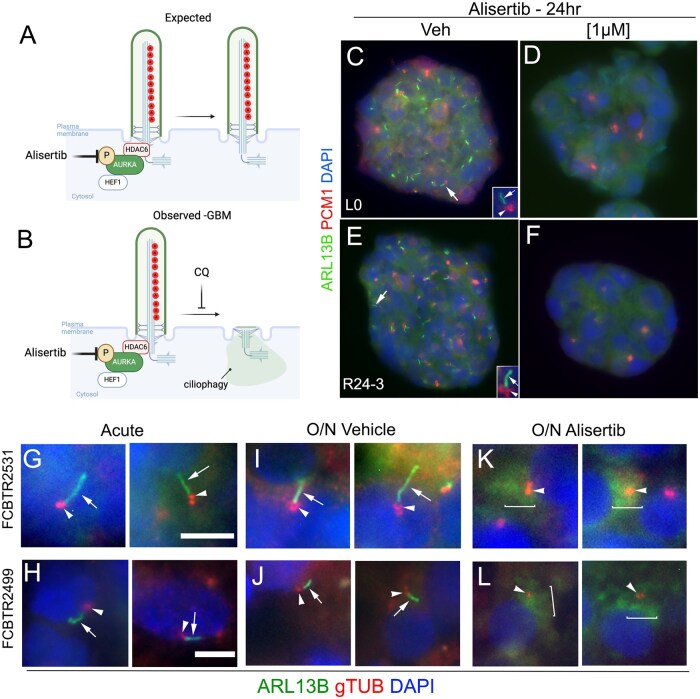
Effect of inhibiting AURKA on GBM primary cilia. (A) Predicted outcome of inhibiting AURKA with Alisertib and preservation of cilia. (B) Observed effect of treating GBM cells with Alisertib, which was significant loss of cilia that could be mitigated partially with chloroquine (CQ). (C-F) Examples of 2 GBM patient gliomaspheres immunolabeled for ARL13B (green, enriched in cilia axoneme) and PCM1 (red, enriched around the basal bodies). The arrows in (C) and (E) spheres point to cilia enlarged in the insets. (G-L) Fresh patient biopsies fixed acutely, or treated overnight with vehicle or Alisertib. Tissues were immunostained for ARL13B (green, arrows) and gTub (red, arrowheads) which labels the ciliary basal body. Nuclei are labeled with DAPI (blue). Scale bars in G and H = 5µm. Figs A, B created with BioRender.com. Figs C-L modified from Ref. 74.

Considering previous studies in normal and other cancer cell types, we expected that treating patient-derived GBM cells with a potent AURKA inhibitor, Alisertib, should stabilize and/or prevent resorption of the cilia (Figure 4A). Instead, we observed that within 24hr of Alisertib treatment, cilia were surprisingly lost (Figure 4B). Figure 4C-F show examples of this phenomenon in two patient-derived cell lines grown as gliomaspheres. Similar observations were made in fresh, surgically resected GBM biopsy material treated ex vivo overnight with Alisertib showed cilia loss (Figure 4G-L) compared to overnight vehicle or acutely fixed specimens. The Alisertib-induced loss of cilia was not observed in cultured primary neurons or glia,^74^ suggesting inhibiting AURKA in GBM cells stimulates a different pathway that drives rapid ciliary disassembly.

In a previous study, our group found that GBM cilia frequency was rapidly reduced in response to alternating electric fields (Tumor Treating Fields) exposure.^75^ Moreover, the loss of cilia was in part mediated by TTFields induction of autophagy, as treatment with the autophagy inhibitor ­chloroquine (CQ) was able to partially prevent the TTFields-induced reduction in cilia. Similarly, we found that CQ mitigated the Alisertib- induced decrease in GBM cilia.^74^ Together these results suggest a ciliophagy mechanism that may be independent of AURKA contributes to the loss of cilia. One possibility is that autophagic stimulation of ciliary disassembly has been shown to go thru HDAC6 activation, although this was not explored.

**Figure 5.**
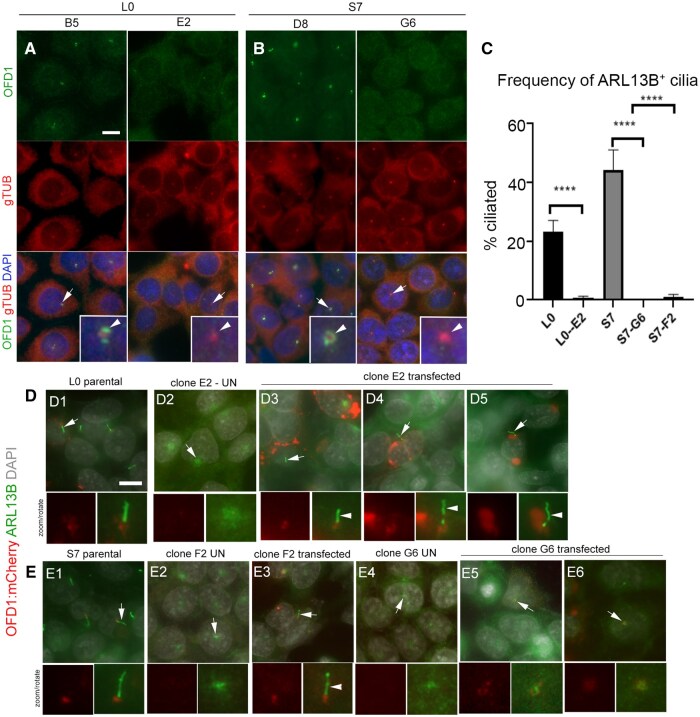
Role for OFD1 in glioma ciliogenesis. (A, B) OFD1 expression in a GBM (L0) and low grade S7 control cell lines. OFD1 (green) normally localizes around the gamma-tubulin (gTub) centrioles (red) (arrowheads in zoom insets of control clones L0-B5 and S7-D8). Note the lack of OFD1 staining around gTub+ centrioles in expanded clones L0-E2 and S7-G6 transfected with CRISPR/Cas9 construct targeting human OFD1. (C) Quantification of cilia frequency in L0, S7 parental (par) and indicated clones. ****P < .001 (ANOVA). (D, E) Transfection of L0 (D1) and S7 (E1) parental and clones with a plasmid expressing OFD1: mcherry which localizes to the cilia basal body in parental cells. Compared to untransfected clones (D2, E2), OFD1-depleted clone L0-E2 (panels D3-D5) and S7-F2 show restored cilia (note endogenous ARL13B) (arrowheads extending from OFD1: mCherry^+^ basal bodies). Cilia were not observed/restored in clone S7-G6 (panels E4-E6). Scale bars in A, D = 5um.

While autophagic-mediated ciliophagy may drive cilia disassembly/loss in some GBM cells, it should be noted that autophagy also has a dual role in promoting ciliogenesis. Activation of autophagy can lead to degradation of Oral-Facial-Digital Syndrome 1(OFD1) protein around the centriolar satellites which promotes ciliogenesis.^76^ Interestingly, a recent study found that OFD1 controls autophagy, and that loss of OFD1 stimulates autophagy. This group also noted that knockout of OFD1 in human kidney 2 (HK2) cells depleted primary cilia, a phenomenon which may be independent of the autophagy activation.^77^ Preliminary studies from our group suggest OFD1 may have a similar role in GBM ciliogenesis. In both high and low grade patient derived glioma lines, we found that depleting OFD1 using CRISPR/Cas9 led to near complete cilia ablation, which could be partially restored by adding back OFD1 to OFD1-depleted cells (Figure 5). Thus, whether loss of cilia is due to enhanced autophagy/ciliophagy or a separate mechanism involving OFD1 requires further investigation.

## Inhibiting vs Overexpressing HDAC6 in Glioma Cells

**Figure 6.**
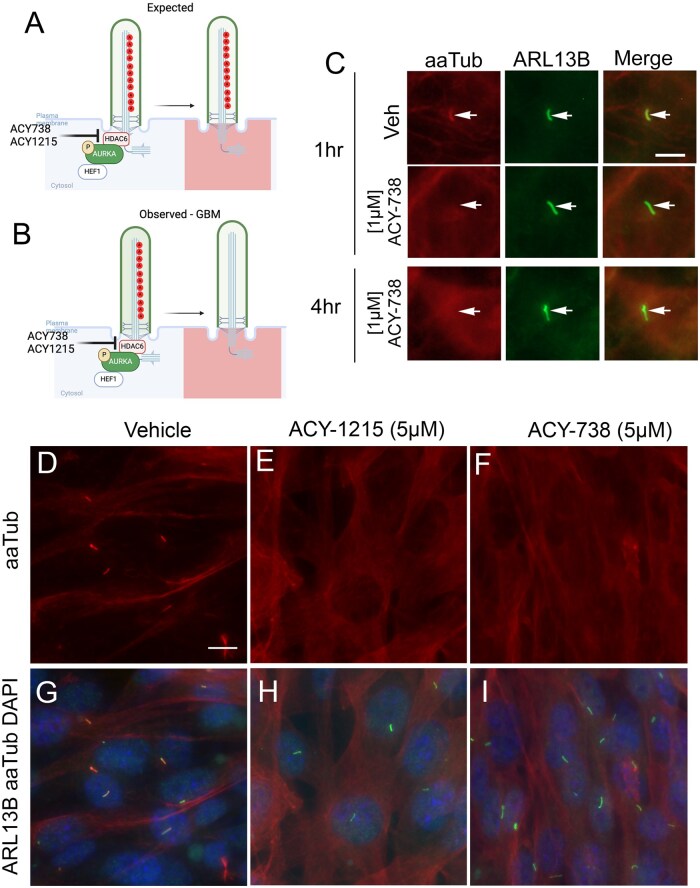
Effect of inhibiting HDAC6 on GBM cilia. (A) Predicted effect that acetylated tubulin+ cilia would persist after various HDAC6 inhibitor (ACY738, ACY1215) treatment. (B) Observed effect of drug-treated GBM cilia that lacked acetylated alpha-tubulin, but displayed enhanced soma signal. (C) Example of GBM patient with loss of aaTUB+ cilia after ACY738. Arrows in Veh show ciliary colocalization of aaTUB (red) or ARL13B (green). (D–I) Treatment of murine KR158 cells with ACY1215 or ACY738 reveal persistence of ARL13B+ cilia that weakly/lack aaTUB. Scale bar in D = 10µm. Figs A, B created with BioRender.com. Figs. C-I modified from Ref. 79.

Not surprisingly, inhibiting HDAC6 in hTERT-RPE1 cells with HDAC inhibitors, such as trichostatin A (TSA) or Tubacin, prevents cilia resorption, increases cilia frequency, and leads to a significant accumulation of acetylated tubulin.^39^ Cilia presumably resorb because the microtubular backbone remains acetylated and stabilized. Indeed in RPE1 cells, inhibition of HDAC6 with Tubacin or Tubastatin A led to increased length and frequencies of aaTUB^+^ cilia.^78^ Thus, we expected that inhibiting HDAC6 in GBM with more recently developed, specific inhibitors would lead to an accumulation of aaTUB^+^ cilia (Figure 6A). While a gross increase in total aaTub protein and cilia frequency were observed, we noted a surprising loss of acetylated tubulin within glioma cilia (Figure 6B).^79^ For example, within 1 hour of treatment with ACY738, we observed ARL13B^+^ cilia membranes are readily detectable but poorly colocalized with aaTUB (Figure 6C). Similarly, in KR158 cells, a murine model of HGG,^80^^,^^81^ ACY1215, or ACY738 induce robust cellular increases in aaTUB but the ARL13B-labeled cilia displayed barely detectable to no aaTUB (Figure 6D-I).

The effect of losing aaTUB in cilia does not appear to be glioma cell-specific. In vitro treatment of normal mouse astrocytes treated with ACY1215 also led to loss of aaTUB in ARL13B^+^ cilia of GFAP^+^ astrocytes.^79^ One possibility is that the use of paraformaldehyde fixation and covalent crosslinking may block acetylated tubulin antibody accessibility within the cilium. However, shortly after publishing our observations in HGG cells, it was reported that genetic ablation of HDAC6 in Zebrafish led to expected increase in cytosolic aaTUB but an unexpected decrease in tubulin acetylation along the axonemes of cilia.^82^ These authors found that HDAC6 inhibitor CAY-10603 also decreased ciliary acetylation. To prepare their samples, they used Dent’s fixative, which is methanol-based.^83^ While this appears to rule out fixation artifacts, it cannot be ruled out that hyperacetylation within cilia disrupts antibody accessibility. Instead, other forms of tubulin modification may be altered or compensating. Indeed, the authors observed increased mono-glycylation but not polyglutamylation in cilia that also showed reduced tubulin acetylation. Thus, in the presence of HDAC6 inhibitors, like other cell types HGG cells may implement other forms of tubulin modifications to ensure axonemal stabilization and function. Interestingly, in U251 and U242 GBM cell lines which display very few cilia, 10 µM Tubastatin A treatment significantly elevated cilia frequencies, which was revealed by IFT88 immunostaining.^84^ Combined with preserved ARL13B expression, these data suggest in the face of HDAC6 inhibition, GBM cells modify their ciliary backbone to preserve some functionality of their cilia. Future studies in GBM cells should examine the various/alternative post-translational modifications of ciliary microtubules.^85^ In addition to acetylation, little is known about glycylation, glutamylation, and detyrosination which would add to our comprehensive understanding of basic or compensating properties of microtubules in the face of HDAC6 or inhibitors.

**Figure 7.**
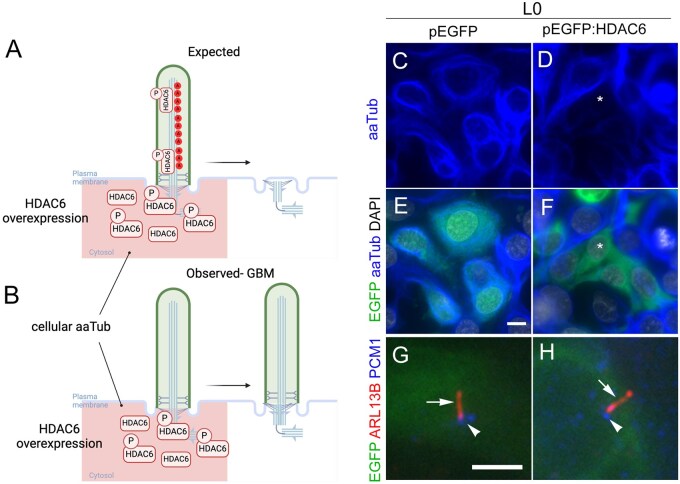
Effect of overexpressing HDAC6 in GBM cells. (A) Predicted effect of cilia loss and cellular reduction in aaTUB after HDAC6 overexpression. (B) Observed effect of persistent cilia in cells with weak cytosolic aaTUB. (C-H) Example of EGFP vs EGFP-HDAC6 overexpression in GBM cell line. Immunostaining of transfected cells for ARL13B (red, arrow) and PCM1+ (blue, arrowhead). EGFP+ cells with HDAC6 overexpression still displayed cilia as shown in (H). Asterisk in (D) shows EGFP-HDAC6+ cells express weak cytosolic aaTUB. Scale bars (in µm) in E = 10 and G = 5. Figs A, B creating with Biorender.com. Figs C-H modified from Ref. 79.

While inhibiting HDAC6 promotes cilia preservation thru unexpected microtubule modifications, overexpressing HDAC6 has been shown to promote cilia loss. For example, in retinal pigmented epithelial 1 (RPE1) cells, overexpressing HA- or GFP-tagged HDAC6 in RPE1 cells resulted in shorter, less frequent aaTUB^+^ cilia.^78^ Similarly, overexpression of flag-tagged HDAC6 reduces aaTUB^+^ cilia frequency of normal cholangiocytes.^86^ Therefore, we expected that overexpression of GFP-tagged HDAC6 in GBM cells to reduce global tubulin acetylation and promote ciliary loss (Figure 7A). As expected, we did observe potent loss of cytosolic aaTub within the transfected cells; however, ARL13B^+^ cilia clearly persisted on these cells (Figure 7C-H). However, HDAC6 overexpression had no effect on cilia length, nor did it reduce ciliary frequency (Figure 7B, G-H).^79^ HDAC6 overexpression even led to increased ciliary frequency in one low-grade glioma line.^79^ While this suggests GFP-tagged HDAC6 retains its enzymatic activity on microtubules in HGG cells, the GFP-fusion was not readily observed within cilia (Figure 7H), which may explain the lack of ciliary changes. However, like RPE1 cells,^78^ we observed endogenous HDAC6 often clustered around basal bodies^79^ although the GFP-fusion also does not seem to cluster around the base, but rather evenly dispersed throughout the cytoplasm. Thus, future studies may need to distinguish whether tagged-HDAC6 or an intrinsic property of ciliary microtubules prevent the tagged-HDAC6 from exerting deacetylatory effects in HGG. Alternatively, HGG cells could maintain cilia that utilize non-HDAC6 as a primary means to disassemble the cilium. Interestingly, Ran et al, identified specific histidine residues in the deacetylase domain of HDAC6, that when mutated to alanine, prevent the deacetylase activity and cilia disassembly.^78^ Thus, given the prominent and reported disproportionate increase in HDAC6 levels in GBM, future studies could examine whether some tumors carry mutations in this domain affecting its ciliary deacetylase capacity and disassembly characteristics. Finally, it should be noted or cautioned that most of these mouse and human glioma studies were performed under 2D rather than 3D conditions which is thought to more closely resemble in vivo characteristics of the tumors and thus requiring further validations.^80^^,^^87^^,^^88^

In summary, our findings in the glioma cell lines tested suggest that inhibiting HDAC6 produces both anticipated and unexpected effects on cilia disassembly. While HDAC6 inhibition appears to prevent cilia loss, this effect does not seem to result from the expected accumulation of tubulin acetylation. Instead, it may involve alternative tubulin modifications occurring within the cilium. These observations indicate a more complex role for HDAC6 inhibition in regulating cilia structure, potentially pointing to compensatory mechanisms that preserve cilia integrity through modifications other than acetylation.

## High Expression of HDAC6, CCRK, VDAC1, LPA, and Many Others Dually Suppress Glioma Cilia Formation and Drive Proliferation

Despite the altered effects of HDAC6 inhibitors on acetylation of glioma cilia (eg using ACY738), we did observe a drug-induced increase in ARL13B^+^ cilia frequency. Interestingly, ACY738-induced increase in glioma cilia was associated with increased differentiation markers and reduced proliferative markers. When ACY738 was applied to glioma cells engineered to lack ARL13B, which ablated the cilia, the inhibitor-induced increase in differentiation and decrease in proliferative markers was not observed.^79^ This key result suggests HDAC6 plays a critical role at cilia to drive proliferation and prevent cells from differentiating.

HDAC6 may be mixed up in a complex of other proteins that cilia glioma cells have exploited to ensure cilia frequency remains low to ensure tumor proliferation. In NIH3T3 fibroblasts induced to disassemble their cilium, knocking down cell cycle related kinase (CCRK) mitigates this process suggesting it may play an active role in cilia disassembly.^89^ CCRK is overexpressed in HGG and knocking it down restored cilia in U251 glioma cells which in turn inhibited their proliferation. Similarly, voltage dependent anion channel 1 (VDAC1) is overexpressed in HGG,^90^ and knockdown of VDAC1 in U87MG or PANC1 cells, a pancreatic cancer cell line, significantly boosted ciliary frequency and decreased cell proliferation.^91^ Whether CCRK and VDAC1 physically interact with HDAC6 at cilia in glioma cells is unclear. Further, while we discuss HDAC6, other HDACs are reported to drive cilia disassembly such as HDAC2^92^ and overexpressed in GBM.^93^ Whether these other HDAC isoforms enhance or promote cilia disassembly and potentiate GBM cell proliferation requires further research.

## Conclusion

GBM cells exhibit a combination of expected and unexpected responses to AURKA and HDAC6 inhibition, raising the question of whether these unpredictable responses confer any advantages to the cells. By bypassing or short-circuiting the core pathway of cilia disassembly, GBM cells may avoid becoming retained in a ciliated or more differentiated state. This alternative mechanism could facilitate rapid ciliary collapse, enabling rapid transformation or proliferation, particularly under stressful environmental conditions or exposure to anti-mitotics. In fact, GBM cells seem to promote ciliogenesis in response to standard of care treatments like temozolomide and irradiation.^32^^,^^75^^,^^94^

The AURKA pathway does not seem to be completely unutilized during cilia disassembly by GBM cells. The oncoprotein BCL2-associated athanogenic protein 3 (BAG3) is another frequently overexpressed protein in GBM.^95^ Recently, it was shown that knocking down BAG3 in U343 and U251 human glioma cells, markedly increased ciliary frequency in these often sparsely ciliated cell lines.^50^ Knocking out BAG3 revealed significant down regulation of AURKA as a top hit by phosphoproteomic analyses of the KO cells, suggesting BAG3 may be driving AURKA activity and maintaining low frequencies of cilia, although a direct interaction between BAG3 and AURKA awaits further biochemical characterization.^50^ BAG3 KO also leads to significant downregulation in NEK2 which may promote ciliary disassembly independent of AURKA. More recently, BAG3 depletion was found to reduce activation of a YAP1/AURKA signaling pathway and induction of PLK1.^84^

The decision to proliferate may not be the only reason GBM cells disassemble its cilium. There may be non-mitotic advantages to ciliary disassembly in GBM cells. For example, in the developing cortical ventricular zone in mice, live imaging of radial glial cells revealed apical excision of their primary cilium at the ventricular surface.^96^ A proposed explanation was to lose responsiveness to sonic hedgehog. More recently, it was found that granule cells of the developing cerebellum permanently disassemble their cilium.^97^ Granule cell progenitors are widely known to mediate SHH to regulate their proliferation, and therefore complete dismantlement of the cilium may be critical for changing or losing responsiveness. Thus, future studies may need to determine if GBM cells disassemble their cilium as a mechanism to change or lose responsiveness local environmental cues.

A consistent feature of GBM is the lower frequency of cilia. Certainly, it is possible many GBMs have aberrantly formed or structurally abnormal cilia,^30^^,^^98^ but more and more proteins within the ciliary disassembly pathway (many listed in Figure 3A) are found to be overexpressed in GBM which may serve as an alternative explanation to lower ciliary frequencies. Like AURKA and HDAC6, NEK2 appears disproportionately highly expressed and promoter of GBM progression.^99^ Since NEK2 activation of Kif24 drives cilia disassembly,^61^ possibly independent of AURKA, one could envision the protein serves to keep ciliary frequencies low in GBM. However, the effects of NEK2 knockdown on GBM cilia remains to be explored. Additional examples include LPA and activation of its receptor LPAR1,^17^^,^^43^^,^^100^ HEF1,^38^^,^^39^ CENPJ,^57^^,^^101^ Cep55,^102^ HIF1a,^103^ Plk1,^104^ calcium,^105^ and Tctex1.^106^ Thus, if cilia are key features to enable tumor cell differentiation, GBM cells may be well equipped to avoid this by upregulating a large array of pathways capable of ensuring disassembly and cell cycle progression.

Understanding whether GBM cells use alternate mechanisms to maintain or modify their cilia could provide insights into how they survive, resist treatment, or evade the body’s immune system. Cilia have been linked to self-renewal or stem-like features on different cell types including GBM.^15^^,^^29^^,^^107^ Preserving the cilium may be key to ensure stem-like properties including quiescence and self-renewal. An intriguing hypothesis that could be investigated is that glioma stem cells and differentiated glioma stem cells use different modes to disassemble their cilium. If such a mechanism exists, there could be future clinical value in arresting glioma stem cells in their ciliated state as an effort to arrest tumor progression. However, simply being arrested in the ciliated state may not be sufficient to halt tumor progression as signaling from the cilium confer pro-tumor properties that require additional clinical targeting to disrupt. For example, ARL13B signaling associated with the cilium appears to be not only important for glioma growth but also angiogenesis and resistance to TMZ and irradiation.^32^^,^^75^^,^^94^^,^^108^ Moreover, recent studies have also observed that immunosuppressive cell types frequently reside in close proximity to GBM cilia and that cilia regulate the release of cytokines which stimulate immunosuppressive cell types to evade immune attack.^109^^,^^110^ Preserving some level of ciliation may thus be advantageous to GBMs as an exploitable safeguard to confer adaptability. As such, a better understanding of these mechanisms may enable innovative therapeutic strategies to disrupt this process and impede disease progression.

Finally, there remain a number of open questions regarding the regulation of cilia disassembly with respect to the onset, progression and treatment resistance in GBM (Box 1). These questions range from cilia dysregulation in the cell of origin to what happens to cilia signaling in the absence or presence of defective cilia. It should be emphasized that primary cilia are recognized mediators of diverse signaling pathways, such as Hedgehog (Hh), receptor tyrosine kinase, PDGF, Wnt, and Notch which have been reviewed extensively.^111^^,^^112^ These pathways are often dysregulated in GBM,^113^ and cilia serve as critical hubs for their regulation. Sitting at the base of cilia is the proteasome, which likely plays a significant role in clearing ubiquitinated structural, and signaling proteins during cilia disassembly. The proteasome relationship to cilia disassembly remains understudied in GBM and is implicated during cancer development.^114^ Collectively, our lack of understanding of how glioma cells break down their primary cilia impacts tumor initiation, progression and treatment response represents a significant unchartered area.

Box 1. Open questions and areas of potential exploration relating to cilia disassembly and GBM onset or progression.

- Does transient or sustained loss or targeting of radial glial/stem cell cilia in adult brain (eg B1 or B2 cells) increase the likelihood of malignant tumors originating from the
- SVZ?
- Are the small population of ciliated tumor cells reported in adult glioma a remnant from radial glial lineage that enable sustained proliferation of the tumors?
- Can GBM be re-capitulated thru forced expression of one or multiple factors associated with cilia disassembly in radial glial stem cell niche in the mature brain?
- Considering the numerous regulators of ciliary disassembly that are often overexpressed in GBM, is it possible to design a multi-inhibitor approach to arrest cells in their ciliated state and inhibit GBM progression?
- Does the sustained loss or suppression of cilia in more differentiated glial cell types, for example mature astrocytes, lead to transformation or ultimately sensitize astrocytes to proliferative cues?
- Cilia mediate and process Hedgehog, Wnt, PDGF, Notch signaling, pathways that are often dysregulated in GBM. Do defects in GBM cilia disassembly change the way these pathways are received and processed to enhance growth or treatment resistance?
- Similar to that reported for LPAR1, do GBM cells that lack, fail to grow or suppress cilia formation re-direct their ciliary signaling machinery to plasma membrane for functional growth advantage?
- Given the low cilia frequency in GBM and widespread increase in HDAC isoforms, could this enhance the rate of ciliary deacetylation, promoting faster cilia disassembly and cell cycle progression?
- How are the components of primary cilia degraded in GBM cells? Does the proteasome function or dysfunction during disassembly promote loss or unstable signaling?

## Data Availability

All data are incorporated into the article.
